# Reevaluation of Gastric Cancer Screening by Levin's Equation

**DOI:** 10.14309/ctg.0000000000000530

**Published:** 2022-09-06

**Authors:** Naoki Ishii, Yasutoshi Shiratori, Takahiko Yano, Mimoe Konai, Yuki Arai, Jun Hamada, Hisato Maekawa, Fumio Omata

**Affiliations:** 1 Division of Gastroenterology, Tokyo Shinagawa Hospital, Tokyo, Japan;; 2 Department of Gastroenterology, Central Hospital of Sherbrooke University, Quebec, Canada;; 3 Division of Gastroenterology, St. Luke's International University, Tokyo, Japan.

## Abstract

This study aimed to reevaluate the effectiveness of fluoroscopy and endoscopy in reducing gastric cancer mortality at the population level.

**METHODS:**

Crude and age-adjusted mortality rates of gastric cancer and the introduction rates of gastric cancer screening were extracted from the Cancer Registry and Statistics database. The population-attributable risk (PAR) percent of no screening for gastric cancer mortality was calculated using Levin's equation. The PAR of each mortality rate in the no-screening group was estimated as follows: mortality × PAR%. The Jonckheere-Terpstra test for trends and linear regression were performed to compare the PAR of gastric cancer mortality rates among the decades.

**RESULTS:**

The PAR of crude and age-adjusted mortality rates in the no-screening group significantly decreased in the total population (*P* for trend <0.001), as well as individually in the male (*P* for trend <0.001) and female (*P* for trend <0.001) populations. The PAR of the crude mortality rate in the female population significantly decreased in 2000–2009 and 2010–2019, compared with that in 1980–1989. There was no significant difference in the PAR of crude mortality rate in the male population among the decades. The PAR of the age-adjusted mortality rate significantly decreased in 2000–2009 and 2010–2019, compared with that in 1980–1989, in the male and female populations.

**DISCUSSION:**

PAR% and PAR of no screening for gastric cancer mortality could be estimated using Levin's equation, and the effectiveness of the present gastric cancer screenings with fluoroscopy and endoscopy has been decreasing, especially in the female population.

## INTRODUCTION

Gastric cancer is an important cause of death in Eastern Asia and for immigrants from high-risk countries such as Asia and South America (1), and is the third leading cause of death in Japan (2). The 5-year survival rate of early gastric cancer, defined as cancer limited to the submucosal layer, is more than 90% (3,4). Although we should consider lead-time bias for cancer survival during cancer screening, early detection and early treatment are important in the management of gastric cancer (5).

There are several gastric cancer screening programs in Japan. The Japan Cancer Society recommends biannual fluoroscopy or endoscopy as the screening modalities for individuals aged 50 years or older (6). Conversely, biannual endoscopy is recommended in the Manual of Gastric Cancer Screening published by the Japanese Society of Gastrointestinal Cancer Screening (7,8). The Japan Research Foundation of Prediction, Diagnosis, and Therapy for Gastric Cancer is focusing on the detection of *Helicobacter pylori* infection and associated gastritis and recommends the ABC method (9,10). The results of the ABC method were divided into 4 groups: group A (*H. pylori* [−], pepsinogen [−]), group B (*H. pylori* [+], pepsinogen [−]), group C (*H. pylori* [+], pepsinogen [+]), and group D (*H. pylori* [−], pepsinogen [+]). Additional endoscopy is recommended for groups B, C, and D for the assessment of gastric cancer. Randomized controlled trials (RCTs) have not been performed to evaluate the efficacy of gastric cancer screening programs at reducing gastric cancer mortality among the 3 groups to date. There are limited data on the effectiveness of the ABC method for mortality reduction, partly because of the more recent introduction of the ABC method. Fluoroscopy and endoscopy screenings are the mainstays of gastric cancer screening programs in Japan.

The proportion of *H. pylori* infection in the Japanese population is gradually decreasing, and the incidence of gastric cancer is expected to decrease in parallel (11). In addition, medical expenditure has been increasing in Japan because of a combination of the rapidly aging society and advanced medical services (12). Because the effectiveness of gastric cancer screening programs with fluoroscopy or endoscopy may decrease at the population level, fluoroscopy or endoscopy screenings targeted for all Japanese people aged 50 years or older are required to be reevaluated as the first-line modalities. This study aimed to reevaluate the effectiveness of fluoroscopy and endoscopy in the reduction of gastric cancer mortality at the population level by estimating the population-attributable risk (PAR) of the crude and age-adjusted mortality in the no-screening population.

## METHODS

### Extraction of population-based data for gastric cancer mortality and detailed dates of gastric cancer screening

Crude and age-adjusted mortality rates of gastric cancer for the Japanese population and the introduction rates of gastric cancer screening were extracted from the Cancer Registry and Statistics database (13). The age-adjusted mortality rate was estimated based on the population-age distributions in 1985. Crude and age-adjusted mortality rates are demonstrated in Supplementary Digital Content (see Supplementary Table 1, http://links.lww.com/CTG/A874). The number of fluoroscopy and endoscopy performed for gastric cancer screening was extracted from e-Stat, a portal site for Japanese Government Statistics (14), and the proportion of endoscopy in the gastric cancer screening was calculated based on the results.

### Estimation of PAR percent of no screening for gastric cancer death using Levin's equation

The population-attributable risk percent (PAR%) is the percent of outcomes that could be prevented if exposures were removed from the population. Basically, the PAR% of gastric cancer mortality was the percent of the no-screening population among gastric cancer deaths. The PAR% of no screening was calculated using Levin's equation (15):

PAR%=exposure prevalence×(relative risk [RR] or odds ratio[OR]−1)/exposure prevalence×[RR or OR−1]+1

No gastric cancer screening (neither fluoroscopy nor endoscopy) was set as the exposure. Participants who underwent gastric cancer screening (fluoroscopy or endoscopy) were set as controls. The exposure prevalence of no screening was calculated as follows: 1 − proportion of participants who underwent gastric cancer screening. Because RR indicated a higher risk of gastric cancer mortality in the no-screening group than in the screening group, the RR in Levin's equation was the inverse number of the RR of gastric cancer mortality reduction by fluoroscopy or endoscopy reported in studies. If the RR of mortality reduction by gastric cancer screening was 0.50 in the article, the RR of gastric cancer mortality was calculated to be 2.0 in the no-screening group compared with that in the screening group.

The PARs of the crude and age-adjusted mortality rates in the no-screening group were estimated as follows: mortality × PAR%.

### Calculating the effectiveness of fluoroscopy or endoscopy screenings for reduction of gastric cancer mortality

There are no RCTs comparing fluoroscopy or endoscopy with no screening for gastric cancer mortality reduction, and a rapid review for PubMed was performed for observational studies on March 20, 2022. At first, systematic reviews of observational studies were searched using the searching terms (see Supplementary Terms, http://links.lww.com/CTG/A874). The latest version of the Japanese Guidelines for Gastric Cancer Screening (7) was also referred to for the assessment of fluoroscopy or endoscopy effectiveness for gastric cancer screening. Backward reference searching was also performed for the adopted papers. Cohort studies in which the follow-up periods were more than 10 years and nested-control studies using large cohorts such as population-based cohorts were adopted for the evaluation of the effectiveness of fluoroscopy and endoscopy screenings.

### Statistical analysis

At first, the PAR% was estimated using Levin's equation according to the introduction rates of gastric cancer screening and the proportion and the number of endoscopy screenings. Second, the PAR% in 2019 was calculated using e-Stat (14) and was applied to the estimation of the PAR.

The Jonckheere-Terpstra test for trends was performed for crude and age-adjusted mortality rates and the PAR of each mortality rate in the no-screening group across decades. Linear regression was performed for the comparison of gastric cancer mortality rates between decades. Because the annual gastric cancer screening program using fluoroscopy was introduced in Japan in the 1980s, and endoscopy including fiberscope has been used for diagnosis after the 1980s (7), the crude and age-adjusted mortality rates in 1980–1989 were referred in the comparison of each mortality across the decades. A 2-tailed *P* value of <0.05 was considered statistically significant. Statistical analyses were performed using STATA version 16 (Stata Corp, College Station, TX). This study was performed using publicly available data. The need to obtain patient informed consent and approval of the Institutional Review Board was waived.

## RESULTS

### Trends and comparisons of crude and age-adjusted mortality in Japan from 1958 to 2019

**Figure 1.**
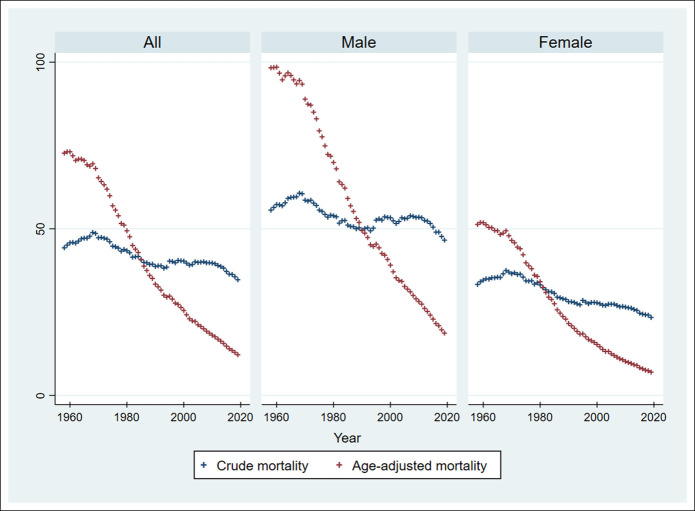
Crude mortality and age-adjusted mortality rates from 1958 to 2019. The Jonckheere-Terpstra test for trends was performed for crude and age-adjusted mortality rates across decades. Crude and age-adjusted mortality significantly decreased in the total population (*P* for trend <0.001 and <0.001), in the male population (*P* for trend <0.001 and <0.001), and in the female population (*P* for trend <0.001 and <0.001). A 2-tailed *P* value of <0.05 was considered statistically significant.

Trends in the crude and age-adjusted mortality rates are shown in Supplementary Digital Content (see Supplementary Table 1, http://links.lww.com/CTG/A874 and Figure 1). Crude and age-adjusted mortality significantly decreased in the total population (*P* for trend <0.001 and <0.001, respectively), in the male population (*P* for trend <0.001 and <0.001, respectively), and in the female population (*P* for trend <0.001 and <0.001, respectively).

The crude and age-adjusted mortality rates were compared across decades, with reference to the mortality rates in 1980–1989 (Table 1). The crude mortality rate in the female population significantly increased in 1958–1969 and 1970–1979 and significantly decreased in 2000–2009 and 2010–2019, compared with that in 1980–1989. However, there was no significant difference in the crude mortality rate for the male population in 2000–2009 and 2010–2019 compared with that in 1980–1989. The age-adjusted mortality rate significantly increased in 1958–1969 and 1970–1979 and significantly decreased in 2000–2009 and 2010–2019 compared with that in 1980–1989 in the male and female populations.

**Table 1.** Comparison of the crude and age-adjusted mortality rate among decades

	Crude mortality rate (number/100,000)					
	All		Male		Female	
	Coefficient, 95% CI	*P* value	Coefficient, 95% CI	*P* value	Coefficient, 95% CI	*P* value
1958–1969	5.64, 4.51 to 6.77	<0.001	6.65, 5.17 to 8.13	<0.001	4.71, 3.80 to 5.61	<0.001
1970–1979	4.55, 3.37 to 5.73	<0.001	4.61, 3.07 to 6.15	<0.001	4.52, 3.57 to 5.47	<0.001
1980–1989	Reference		Reference		Reference	
1990–1999	−1.57, −2.75 to 0.39	0.010	−0.20, −1.74 to 1.34	0.796	−2.80, −3.75 to 1.85	<0.001
2000–2009	−1.20, −2.38 to 0.02	0.047	1.32, −0.22 to 2.86	0.092	−3.47, −4.42 to 2.52	<0.001
2010–2019	−3.48, −4.66 to 2.30	<0.001	−1.08, −2.62 to 0.46	0.167	−5.55, −6.50 to 4.60	<0.001

	Age-adjusted mortality rate (number/100,000)					
	All		Male		Female	
	Coefficient, 95% CI	*P* value	Coefficient, 95% CI	*P* value	Coefficient, 95% CI	*P* value
1958–1969	29.07, 26.21 to 31.92	<0.001	35.58, 31.94 to 39.22	<0.001	21.99, 19.82 to 24.15	<0.001
1970–1979	16.66, 13.68 to 19.64	<0.001	20.37, 16.57 to 24.17	<0.001	13.13, 10.87 to 15.39	<0.001
1980–1989	Reference		Reference		Reference	
1990–1999	−11.97, −14.95 to 8.99	<0.001	−15.30, −19.10 to 11.50	<0.001	−9.48, −11.74 to 7.22	<0.001
2000–2009	−19.98, −22.96 to 17.00	<0.001	−26.88, −30.68 to 23.08	<0.001	−15.24, −17.50 to 12.98	<0.001
2010–2019	−26.51, −29.49 to 23.53	<0.001	−36.89, −40.69 to 33.09	<0.001	−19.40, −21.66 to 17.14	<0.001

Linear regression was performed for the comparison of crude and age-adjusted mortality among the decades. A 2-tailed *P* value of <0.05 was considered statistically significant.

CI, confidence interval.

### Effectiveness of gastric cancer screenings with fluoroscopy or endoscopy in mortality reduction

Overall, 127 papers were searched. Zhang et al. previously published a meta-analysis evaluating the effectiveness of endoscopy screening (16). There were no published systematic reviews or meta-analysis of fluoroscopy effectiveness. The effectiveness of fluoroscopy and endoscopy screenings for mortality reduction is demonstrated in Table 2. The effectiveness of gastric cancer screening was different among the studies (7,16–22). The effectiveness of fluoroscopy screening was determined to be ∼50% (RR 0.50) in 2 cohort studies in which follow-up periods were over 10 years. The effectiveness of endoscopy screening was 0.58 in the meta-analysis and ranged from 0.19 to 0.60 depending on the number of endoscopies in the nested case-control study using a population-based cohort (22): 1 endoscopy, RR 0.60, 40% reduction; 2 endoscopies, RR 0.32, 68% reduction; and 3 or more endoscopies, RR 0.19, 81% reduction.

**Table 2.** Effectiveness of gastric cancer screening with fluoroscopy or endoscopy for gastric cancer mortality reduction

Authors	Publication year	Modality	No. of subjects	Study periods	Follow-up periods	RR or OR for mortality (95% confidence interval)
Inaba S (17)	1999	Fluoroscopy	Screened 9,142 Not screened 14,992	1992–1995	40 mo	Male 0.72 (0.31–1.66) Female 1.46 (0.43–4.90)
Mizoue T (18)	2003	Fluoroscopy	Screened 30,771 Not screened 56,541	1988–1997	Mean 8 yr	Male 0.65 (0.45–0.95) Female 0.75 (0.42–1.34)
Lee KJ (19)	2006	Fluoroscopy	Screened 15,189 Not screened 26,961	1990–2003	13 yr	0.52 (0.36–0.78)
Miyamoto A (20)	2007	Fluoroscopy	Screened 24,014 Not screened 17,380	1990–2001	11 yr	0.54 (0.38–0.77)
Hosokawa O (21)	2008	Endoscopy	Screened 2,192 Not screened 9,571	Entry, 1990–1992	10 yr	0.35 (0.14–0.86)
Jun JK (22)	2017	Endoscopy	Nested case-control study Case 54,418 Control 217,672	2002–2012	11 yr Population-based cohort	All 0.53 (0.51–0.56) Once 0.60 (0.57–0.63) Twice 0.32 (0.28–0.37) 3 or more 0.19 (0.14–0.26)
Zhang X (16)	2018	Endoscopy	342,013, all from Asia	Meta-analysis of 6 cohort studies and 4 nested case-control studies		0.58 (0.48–0.70)

The effectiveness of gastric cancer screenings was different among the studies. The effectiveness of fluoroscopy screening for gastric cancer mortality reduction was considered about 50% (RR 0.50) in the cohort studies in which follow-up periods were over 10 years. The effectiveness of endoscopy screening for gastric cancer mortality reduction was 0.58 in the meta-analysis (16) and ranged from 0.19 to 0.60 depending on the number of endoscopies in the nested case-control study (22).

RR, relative risk.

Expected mortality reduction by screening and RR of no screening for gastric cancer mortality are demonstrated according to the effectiveness and proportion of endoscopy screening in the Supplementary Digital Content (see Supplementary Table 2, http://links.lww.com/CTG/A874). The expected mortality reduction in gastric cancer screening ranged from 0.20 (80% mortality reduction) to 0.60 (40% mortality reduction). The RR of no screening to screening was estimated to range from 1.67 to 5.00.

### PAR% of no screening for gastric cancer mortality estimated using Levin's equation

The PAR% of no screening was estimated using Levin's equation according to the introduction rates of gastric cancer screening and the proportion and the number of endoscopy screenings (Table 3). The number of endoscopy screenings contributed to mortality reduction (22), and the PAR% of no screening increased according to the number of endoscopy screenings. Because more than 2 endoscopy screenings were considered more effective than fluoroscopy screening (1 endoscopy, RR 0.6; 2 endoscopies, RR ∼0.3 [0.32]; 3 or more endoscopies, RR ∼0.2 [0.19]; fluoroscopy, RR ∼0.5 [0.52–0.54]), the PAR% of no screening for gastric cancer death increased according to the increased proportion of endoscopy screening in the 2 and ≥3 endoscopy groups. The higher rates of the introduction of gastric cancer screening were associated with lower PAR% of no screening.

**Table 3.** PAR% of no screening for gastric cancer death by Levin's equation

		Proportion of endoscopy screening, 3 or more endoscopy screenings										
		0.00	0.10	0.20	0.30	0.40	0.50	0.60	0.70	0.80	0.90	1.00
Introduction rate of gastric cancer screening	0.10	0.47	0.50	0.53	0.56	0.59	0.63	0.66	0.69	0.72	0.75	0.78
	0.20	0.44	0.47	0.50	0.54	0.57	0.60	0.63	0.66	0.69	0.73	0.76
	0.30	0.41	0.44	0.47	0.50	0.53	0.57	0.60	0.63	0.67	0.70	0.74
	0.40	0.38	0.40	0.43	0.46	0.49	0.53	0.56	0.59	0.63	0.67	0.71
	0.50	0.33	0.36	0.39	0.42	0.45	0.48	0.52	0.55	0.59	0.63	0.67
	0.60	0.29	0.31	0.34	0.37	0.39	0.43	0.46	0.49	0.53	0.57	0.62
	0.70	0.23	0.25	0.28	0.30	0.33	0.36	0.39	0.42	0.46	0.50	0.55
	0.80	0.17	0.18	0.20	0.22	0.25	0.27	0.30	0.33	0.36	0.40	0.44
	0.90	0.09	0.10	0.11	0.13	0.14	0.16	0.18	0.20	0.22	0.25	0.29

		Proportion of endoscopy screening, 2 endoscopy screenings										
		0.00	0.10	0.20	0.30	0.40	0.50	0.60	0.70	0.80	0.90	1.00
Introduction rate of gastric cancer screening	0.10	0.47	0.49	0.51	0.53	0.55	0.57	0.59	0.62	0.64	0.66	0.68
	0.20	0.44	0.46	0.48	0.50	0.52	0.55	0.57	0.59	0.61	0.63	0.65
	0.30	0.41	0.43	0.45	0.47	0.49	0.51	0.53	0.55	0.58	0.60	0.62
	0.40	0.38	0.39	0.41	0.43	0.45	0.47	0.49	0.52	0.54	0.56	0.58
	0.50	0.33	0.35	0.37	0.39	0.41	0.43	0.45	0.47	0.49	0.52	0.54
	0.60	0.29	0.30	0.32	0.34	0.36	0.38	0.39	0.42	0.44	0.46	0.48
	0.70	0.23	0.25	0.26	0.28	0.29	0.31	0.33	0.35	0.37	0.39	0.41
	0.80	0.17	0.18	0.19	0.20	0.22	0.23	0.25	0.26	0.28	0.30	0.32
	0.90	0.09	0.10	0.11	0.11	0.12	0.13	0.14	0.15	0.16	0.18	0.19

		Proportion of endoscopy screening, 1 endoscopy screening										
		0.00	0.10	0.20	0.30	0.40	0.50	0.60	0.70	0.80	0.90	1.00
Introduction rate of gastric cancer screening	0.10	0.47	0.46	0.45	0.44	0.43	0.42	0.41	0.40	0.39	0.38	0.38
	0.20	0.44	0.43	0.42	0.42	0.41	0.40	0.39	0.38	0.37	0.36	0.35
	0.30	0.41	0.40	0.39	0.38	0.37	0.36	0.35	0.35	0.34	0.33	0.32
	0.40	0.38	0.37	0.36	0.35	0.34	0.33	0.32	0.31	0.30	0.29	0.29
	0.50	0.33	0.32	0.32	0.31	0.30	0.29	0.28	0.27	0.27	0.26	0.25
	0.60	0.29	0.28	0.27	0.26	0.25	0.25	0.24	0.23	0.22	0.22	0.21
	0.70	0.23	0.22	0.22	0.21	0.20	0.20	0.19	0.18	0.18	0.17	0.17
	0.80	0.17	0.16	0.16	0.15	0.15	0.14	0.14	0.13	0.13	0.12	0.12
	0.90	0.09	0.09	0.08	0.08	0.08	0.08	0.07	0.07	0.07	0.06	0.06

PAR% of no screening for gastric cancer death was calculated using Levin's equation.

PAR% = exposure prevalence × (RR [or OR] − 1)/{exposure prevalence × (RR [or OR] − 1) + 1}.

No gastric cancer screening (fluoroscopy or endoscopy) was set as the exposure.

Subjects who underwent gastric cancer screening (fluoroscopy or endoscopy) were set as control groups.

The RR indicated a higher risk for gastric cancer mortality in the no-screening group compared with the screening group.

The RRs of the no-screening group compared with the screening group for gastric cancer death were estimated by the effectiveness and the proportion of fluoroscopy and endoscopy screenings and demonstrated in Supplementary Digital Content (see Supplementary Table 2, http://links.lww.com/CTG/A874).

The PAR% of no screening for gastric cancer death changes according to the introduction rate of gastric cancer screening, the proportion of endoscopy screening, and the number of endoscopy screenings.

OR, odds ratio; PAR%, population-attributable risk percent; RR, relative risk.

The PAR% in 2019 was calculated using Levin's equation and the e-Stat data (14) (Table 4). The introduction rate of gastric cancer screening for those aged 50 years or older within 2 years was 0.45 in the total population, 0.50 in the male population, and 0.40 in the female population in 2019 (13). The screening information from local communities was not complete in the e-Stat. However, the proportion of endoscopy screening in gastric cancer screening could be estimated by the number of screening fluoroscopies and endoscopies performed in 2019 according to the e-Stat (14): 0.30 in the total population, 0.29 in the male population, and 0.31 in the female population. The PAR% of no screening in 2019 ranged from 0.31 to 0.47 (Table 4) and was used for the PAR estimation.

**Table 4.** PAR% of no screening for gastric cancer death in 2019 estimated using Levin's equation

	Introduction rate of gastric cancer screening	Fluoroscopy, n	Endoscopy, n	Proportion of endoscopy	PAR% (RR 0.2)	PAR% (RR 0.3)	PAR% (RR 0.6)
All	0.45	2,586,791	1,111,277	0.30	0.44	0.41	0.33
Male	0.50	1,155,765	468,435	0.29	0.42	0.39	0.31
Female	0.40	1,431,026	642,842	0.31	0.47	0.43	0.35

The introduction rate of gastric cancer screening for those aged 50 years or older within 2 years was 0.45 in the total population, 0.50 in the male population, and 0.40 in the female population in 2019 (13). The screening information from local communities was not complete in the e-Stat (14). However, the proportion of endoscopy screening in gastric cancer screening could be estimated by the number of fluoroscopy and endoscopy performed for screening in 2019 according to the data of e-Stat. PAR% of no screening for gastric cancer death in 2019 was calculated using Levin's equation in the RR of endoscopy screening 0.2, 0.3, and 0.6.

PAR%, population-attributable risk percent; RR, relative risk.

### PAR of crude and age-adjusted mortality rates in the no-screening population

**Figure 2.**
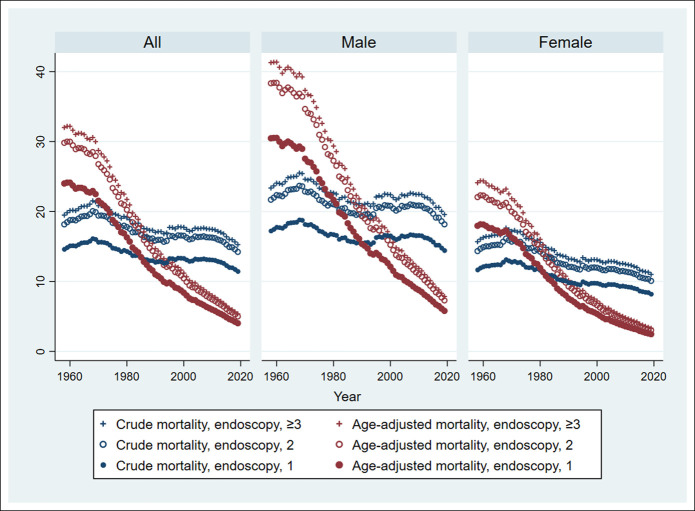
Estimated PAR of crude and age-adjusted mortality rates of no screening from 1958 to 2019. The Jonckheere-Terpstra test for trends was performed for the PAR of crude and age-adjusted mortality rates of no screening. The PAR of crude and age-adjusted mortality rates for no screening significantly decreased in the total population (*P* for trend <0.001), in the male population (*P* for trend <0.001), and in the female population (*P* for trend <0.001), irrespective of the number of endoscopy screenings. A 2-tailed *P* value of <0.05 was considered statistically significant. PAR, population-attributable risk.

The PAR of the crude and age-adjusted mortality rates in the no-screening population was estimated by (crude or age-adjusted mortality rates) × (PAR% of no screening in 2019) and is shown in Figure 2. The PAR of crude and age-adjusted mortality rates significantly decreased in the total population (*P* for trend <0.001), in the male population (*P* for trend <0.001), and in the female population (*P* for trend <0.001), irrespective of the number of endoscopy screenings.

The PAR of the crude and age-adjusted mortality rates were compared among the decades, referred to as the mortality rates in 1980–1989 (Table 5). The PAR of the crude mortality rate in the female population significantly increased in 1958–1969 and 1970–1979 and significantly decreased in 2000–2009 and 2010–2019, compared with that in 1980–1989, irrespective of the number of endoscopy screenings. There was no significant difference in the PAR of the crude mortality rate in the male population in 2000–2009 and 2010–2019, compared with that in 1980–1989. The PAR of the age-adjusted mortality rate significantly increased in 1958–1969 and 1970–1979 and significantly decreased in 2000–2009 and 2010–2019, compared with that in 1980–1989, in the male and female populations.

**Table 5.** Comparison of the crude and age-adjusted mortality rate of the no-screening population estimated using Levin's equation

	Three or more endoscopy screenings							Three or more endoscopy screenings					
	Crude mortality rate (number/100,000)							Age-adjusted mortality rate (number/100,000)					
	All		Male		Female			All		Male		Female	
	Coefficient, 95% CI	*P* value	Coefficient, 95% CI	*P* value	Coefficient, 95% CI	*P* value		Coefficient, 95% CI	*P* value	Coefficient, 95% CI	*P* value	Coefficient, 95% CI	*P* value
1958–1969	2.48, 1.99 to 2.98	<0.001	2.79, 2.17 to 3.41	<0.001	2.21, 1.79 to 2.64	<0.001	1958–1969	12.79, 11.54 to 14.04	<0.001	14.93, 13.41 to 16.46	<0.001	10.33, 9.31 to 11.36	<0.001
1970–1979	2.01, 1.49 to 2.53	<0.001	1.94, 1.29 to 2.59	<0.001	2.13, 1.68 to 2.57	<0.001	1970–1979	7.33, 6.02 to 8.64	<0.001	8.55, 6.95 to 10.14	<0.001	6.17, 5.10 to 7.23	<0.001
1980–1989	Reference		Reference		Reference		1980–1989	Reference		Reference		Reference	
1990–1999	−0.68, −1.20 to 0.16	0.011	−0.08, −0.73 to 0.57	0.812	−1.32, −1.77 to 0.88	<0.001	1990–1999	−5.27, −6.58 to 3.96	<0.001	−6.43, −8.02 to 4.83	<0.001	−4.45, −5.52 to 3.39	<0.001
2000–2009	−0.53, −1.05 to 0.01	0.047	0.55, −0.10 to 1.20	0.093	−1.63, −2.08 to 1.19	<0.001	2000–2009	−8.79, −10.10 to 7.49	<0.001	−11.30, −12.89 to 9.70	<0.001	−7.16, −8.22 to 6.09	<0.001
2010–2019	−1.53, −2.05 to 1.01	<0.001	−0.45, −1.10 to 0.20	0.169	−2.61, −3.05 to 2.16	<0.001	2010–2019	−11.67, −12.98 to 10.36	<0.001	−15.49, −17.09 to 13.90	<0.001	−9.11, −10.18 to 8.05	<0.001

	Two endoscopy screenings							Two endoscopy screenings					
	Crude mortality rate (number/100, 000)							Age-adjusted mortality rate (number/100, 000)					
	All		Male		Female			All		Male		Female	
	Coefficient, 95% CI	*P* value	Coefficient, 95% CI	*P* value	Coefficient, 95% CI	*P* value		Coefficient, 95% CI	*P* value	Coefficient, 95% CI	*P* value	Coefficient, 95% CI	*P* value
1958–1969	2.32, 1.85 to 2.78	<0.001	2.59, 2.01 to 3.17	<0.001	2.03, 1.64 to 2.42	<0.001	1958–1969	11.92, 10.75 to 13.08	<0.001	13.87, 12.45 to 15.29	<0.001	9.46, 8.53 to 10.39	<0.001
1970–1979	1.87, 1.39 to 2.36	<0.001	1.80, 1.20 to 2.40	<0.001	1.95, 1.54 to 2.35	<0.001	1970–1979	6.83, 5.61 to 8.05	<0.001	7.94, 6.46 to 9.42	<0.001	5.64, 4.67 to 6.62	<0.001
1980–1989	Reference		Reference		Reference		1980–1989	Reference		Reference		Reference	
1990–1999	−0.64, −1.12 to 0.15	0.011	−0.07, −0.67 to 0.53	0.812	−1.21, −1.62 to 0.80	<0.001	1990–1999	−4.91, −6.13 to 3.69	<0.001	−5.97, −7.45 to 4.49	<0.001	−4.07, −5.05 to 3.10	<0.001
2000–2009	−0.49, −0.98 to 0.01	0.047	0.51, −0.09 to 1.12	0.093	−1.49, −1.90 to 1.09	<0.001	2000–2009	−8.20, −9.41 to 6.98	<0.001	−10.49, −11.97 to 9.01	<0.001	−6.55, −7.52 to 5.58	<0.001
2010–2019	−1.42, −1.91 to 0.94	<0.001	−0.42, −1.02 to 0.18	0.169	−2.38, −2.79 to 1.98	<0.001	2010–2019	−10.87, −12.09 to 9.65	<0.001	−14.39, −15.87 to 12.90	<0.001	−8.34, −9.31 to 7.36	<0.001

	One endoscopy screening							One endoscopy screening					
	Crude mortality rate (number/100, 000)							Age-adjusted mortality rate (number/100, 000)					
	All		Male		Female			All		Male		Female	
	Coefficient, 95% CI	*P* value	Coefficient, 95% CI	*P* value	Coefficient, 95% CI	*P* value		Coefficient, 95% CI	*P* value	Coefficient, 95% CI	*P* value	Coefficient, 95% CI	*P* value
1958–1969	1.86, 1.49 to 2.24	<0.001	2.06, 1.60 to 2.52	<0.001	1.65, 1.33 to 1.97	<0.001	1958–1969	9.59, 8.65 to 10.53	<0.001	11.02, 9.90 to 12.15	<0.001	7.70, 6.94 to 8.46	<0.001
1970–1979	1.51, 1.12 to 1.90	<0.001	1.43, 0.95 to 1.91	<0.001	1.58, 1.25 to 1.92	<0.001	1970–1979	5.50, 4.52 to 6.48	<0.001	6.31, 5.13 to 7.49	<0.001	4.59, 3.80 to 5.39	<0.001
1980–1989	Reference		Reference		Reference		1980–1989	Reference		Reference		Reference	
1990–1999	−0.51, −0.90 to 0.12	0.011	−0.06, −0.54 to 0.42	0.812	−0.98, −1.32 to 0.65	<0.001	1990–1999	−3.95, −4.93 to 2.97	<0.001	−4.74, −5.92 to 3.57	<0.001	−3.31, −4.11 to 2.52	<0.001
2000–2009	−0.40, −0.79 to 0.01	0.047	0.41, −0.07 to 0.89	0.093	−1.22, −1.55 to 0.88	<0.001	2000–2009	−6.60, −7.58 to 5.61	<0.001	−8.34, −9.52 to 7.16	<0.001	−5.33, −6.12 to 4.54	<0.001
2010–2019	−1.14, −1.54 to 0.75	<0.001	−0.33, −0.81 to 0.15	0.169	−1.94, −2.27 to 1.61	<0.001	2010–2019	−8.75, −9.73 to 7.77	<0.001	−11.43, −12.61 to 10.26	<0.001	−6.78, −7.58 to 5.99	<0.001

Linear regression was performed for the comparison of the decades in each mortality.

PAR% was calculated using Levin's equation.

The proportions of endoscopy screening were set as follows: 30% in all, 29% in males, and 31% in females.

The PARs% of no screening were as follows: 3 or more endoscopy screenings: 44% in all, 42% in males, and 46% in females; 2 endoscopy screenings: 41% in all, 39% in males, and 43% in females; 1 endoscopy screening: 33% in all, 31% in males, and 35% in females.

A 2-tailed *P* value of <0.05 was considered statistically significant.

CI, confidence interval; PAR%, population-attributable risk percent.

## DISCUSSION

This study was a program reevaluation study for gastric cancer screenings with fluoroscopy or endoscopy. The PAR% of the no-screening population for gastric cancer death was estimated using Levin's equation, based on the introduction rates of gastric cancer screening and the proportion of endoscopy screening. The PAR of crude and age-adjusted mortality rates has been significantly decreasing for decades in the no screening of the female population group, and gastric cancer screening with fluoroscopy or endoscopy targeted for all populations aged 50 years or older should be reconsidered.

The incidence and mortality of gastric cancer differ between countries and among immigrants and native populations within countries. Japan is one of the countries with the highest incidence and mortality rates worldwide (1). However, the population-level effectiveness of gastric cancer screening programs is expected to decrease according to the decreased burden of gastric cancer in Japan (2), and we should therefore reevaluate the effectiveness of fluoroscopy and endoscopy programs at the population level. A nested case-control study demonstrated that endoscopy screening reduced gastric cancer mortality (22). However, the attributable risk of gastric cancer mortality, the population-level evidence, could not be obtained in the study (22). The effectiveness of endoscopic screening has been previously reported in a systematic review and meta-analysis in which the effectiveness of endoscopy was compared with that of no screening (RR, 0.58; 95% confidence interval, 0.48–0.70) (16). Therefore, gastric cancer screening by endoscopy was effective at an individual level for opportunistic screening, but sufficient evidence was not obtained as a population-based screening. The risk differences between screened and unscreened populations, rather than RR or OR, are important in the application of obtained evidence at the population level (23). RCTs and cohort studies cannot be performed many times to evaluate risk differences in mortality rates between screening and no-screening groups across decades. In this study, the PAR% of no screening was estimated using Levin's equation, referring to the introduction rates of gastric cancer screening and the proportion of endoscopy screening, and the trends of PAR of crude and age-adjusted mortality rates were evaluated in no-screening populations.

Although there may be several reasons to explain the decreasing trends of gastric cancer mortality, including the advancement of endoscopic technologies (3,4,24), the most important cause of the decreasing burden of gastric cancer is considered to be the decreasing prevalence of *H. pylori* infection (11). *H. pylori* has been considered an important causal factor driving the development of gastric cancer since 1994, when the World Health Organization/International Agency for Research on Cancer defined *H. pylori* as a definite carcinogen (25). The risk of gastric cancer development varies greatly between *H. pylori*–positive and *H. pylori*–negative populations, and a prior cohort study from Japan demonstrated that gastric cancer developed only in *H. pylori*–positive patients, but not in uninfected patients (26). A prospective study from a Japanese population also demonstrated that dietary salt intake was a risk factor for gastric cancer (27). In this study, a significant association between salt intake and gastric cancer was observed only in patients with *H. pylori* infection and atrophic gastritis. *H. pylori* infection and associated gastritis were considered important in this study. Although the development of gastric cancers in *H. pylori*–negative subjects has recently been reported, the proportion of *H. pylori*–negative gastric cancers was only 1.2% of all gastric cancers, and the clinical courses of *H. pylori*–negative gastric cancers have not been fully elucidated (28). From the standpoint of public health, the benefits of gastric cancer screening programs in *H. pylori*–negative populations are very small at the population level. *H. pylori* infection should be considered as the most important causal factor, and atrophic gastritis mostly caused by *H. pylori* infection can be considered as a mediator between *H. pylori* infection and the development of gastric cancer. We need to include *H. pylori* and associated gastritis in gastric cancer screening programs. The PAR of the crude and the age-adjusted mortality rate has been significantly decreasing in the female population, and the prevalence of *H. pylori* infection and associated gastritis may be lower in the female population.

The sensitivity and the specificity of *H. pylori* antibody are ∼80%; thus, only *H. pylori* antibody was not desirable as a single screening method for *H. pylori* infection diagnosis (29). The sensitivity and the specificity of the stool antigen test and urea breath test were excellent, with a reported efficacy of 92%–95% (30). There are several combined screening methods, and a simultaneous screening program is superior to single screening and sequential screening for increasing net sensitivity (31). Considering the high sensitivity of both tests (urea breath test and stool antigen test), but the complexity of the urea breath test, the ABC method (combination of *H. pylori* antibody and pepsinogen, a substitute for atrophic gastritis, a mediator between *H. pylori* and gastric cancer) combined with the stool antigen test could be a desirable method for the detection of *H. pylori* infection and atrophic gastritis. Endoscopy following the tests for *H. pylori* infection and atrophic gastritis can be the sequential screening in the gastric cancer screening programs.

The strength is that we were able to estimate the PAR% of no screening for gastric cancer mortality using Levin's equation. In addition, to the best of our knowledge, this study is the first report to evaluate the trends of the PAR of crude and age-adjusted mortality in the no-screening population by fluoroscopy or endoscopy.

However, this study has several limitations. First, this study was not an RCT, and confounders could not be eliminated to evaluate screening effectiveness. Second, risk differences and population-level effectiveness were not evaluated across the decades between the screening and no-screening groups. Finally, the PAR of no screening for gastric cancer mortality was not calculated based on detailed patient data. However, the results can contribute to the introduction and advancement of gastric cancer programs in other countries.

In conclusion, the PAR% and PAR of no screening for gastric cancer mortality could be estimated using Levin's equation. This evaluation revealed that the effectiveness of the present gastric cancer screenings with fluoroscopy and endoscopy has been decreasing, especially in the female population. These results indicate that the important causal factor, *H. pylori* infection and associated gastritis, should be included in gastric cancer screening programs to increase effectiveness.

## CONFLICTS OF INTEREST

**Guarantor of the article:** Naoki Ishii, MD, PhD, MPH.

**Specific author contributions:** All the authors have contributed significantly to and agree on the content of the manuscript. Each author's contribution to the manuscript is as follows: N.I.: designed and conducted the study and performed statistical analysis; N.I., Y.S., and F.O.: collected and interpreted the data; N.I.: drafted the article; Y.S., T.Y., M.K., Y.A., H.M., and F.O.: critically revised the manuscript. All authors read and approved the submitted version of the manuscript.

**Financial support:** None to report.

**Potential competing interests:** None to report.

**Study Highlights**

**WHAT IS KNOWN**

✓ The effectiveness of gastric cancer screening programs with fluoroscopy or endoscopy may decrease at the population level.
✓ Fluoroscopy or endoscopy screenings are required to be reevaluated as the first-line modalities.

**WHAT IS NEW HERE**

✓ The population-attributable risk percent and population-attributable risk of no screening for gastric cancer death could be estimated using Levin's equation.
✓ The effectiveness of the present gastric cancer screenings with fluoroscopy and endoscopy has been decreasing, especially in the female population.

## Supplementary Material

SUPPLEMENTARY MATERIAL
