# Supplementary material for: Reevaluation of Gastric Cancer Screening by Levin's Equation
Source: Clin Transl Gastroenterol. 2022 Sep 6;13(10):e00530. doi: 10.14309/ctg.0000000000000530 (PMC9624491; doi:10.14309/ctg.0000000000000530)
Supplement: SUPPLEMENTARY MATERIAL [file ct9-13-e00530-s001.docx]

**Searching terms**

(("Stomach Neoplasms"[MeSH Terms] OR (("gastric"[Title/Abstract] OR "stomach"[Title/Abstract]) AND ("cancer*"[Title/Abstract] OR "neoplasm*"[Title/Abstract] OR "tumor*"[Title/Abstract] OR "tumour*"[Title/Abstract]))) AND ((("upper gastrointestinal series"[All Fields] OR “barium”[MeSH Terms] OR fluoroscopy[All Fields] OR x-ray[all Fields]) OR ("endoscopie"[All Fields] OR "endoscopy"[MeSH Terms] OR "endoscopy"[All Fields] OR "endoscopies"[All Fields] OR "endoscopy s"[All Fields])) AND ("diagnosis"[MeSH Subheading] OR "diagnosis"[All Fields] OR "screening"[All Fields] OR "mass screening"[MeSH Terms] OR ("mass"[All Fields] AND "screening"[All Fields]) OR "mass screening"[All Fields] OR "early detection of cancer"[MeSH Terms] OR ("early"[All Fields] AND "detection"[All Fields] AND "cancer"[All Fields]) OR "early detection of cancer"[All Fields] OR "screen"[All Fields] OR "screenings"[All Fields] OR "screened"[All Fields] OR "screens"[All Fields]))) AND (systematicreview[Filter]).

**Supplementary Table 1. Crude and age-adjusted mortality of gastric cancer in Japan from 1958 to 2019**

| **ICD** | **ICD code** | **Year** | **Crude mortality rate  (/100,000)** | | | **Age-adjusted mortality rate (/100,000)** | | |
| --- | --- | --- | --- | --- | --- | --- | --- | --- |
|  |  |  | **All** | **Male** | **Female** | **All** | **Male** | **Female** |
| ICD-7 | 151 | 1958 | 44.3 | 55.6 | 33.3 | 72.7 | 98.3 | 51.3 |
| ICD-7 | 151 | 1959 | 45.1 | 56.4 | 34.1 | 73.1 | 98.4 | 51.9 |
| ICD-7 | 151 | 1960 | 45.8 | 57.3 | 34.6 | 73.1 | 98.5 | 51.8 |
| ICD-7 | 151 | 1961 | 45.9 | 57.2 | 35.0 | 71.9 | 96.7 | 51.2 |
| ICD-7 | 151 | 1962 | 45.7 | 56.9 | 34.9 | 70.5 | 94.7 | 50.4 |
| ICD-7 | 151 | 1963 | 46.3 | 57.8 | 35.3 | 70.9 | 95.9 | 50.3 |
| ICD-7 | 151 | 1964 | 47.0 | 59.1 | 35.3 | 70.9 | 96.8 | 49.5 |
| ICD-7 | 151 | 1965 | 47.2 | 59.4 | 35.5 | 70.5 | 96.0 | 49.4 |
| ICD-7 | 151 | 1966 | 47.2 | 59.5 | 35.4 | 69.2 | 94.7 | 48.3 |
| ICD-7 | 151 | 1967 | 47.8 | 59.6 | 36.5 | 68.8 | 93.5 | 48.7 |
| ICD-8 | 151 | 1968 | 48.9 | 60.7 | 37.5 | 69.5 | 94.5 | 49.4 |
| ICD-8 | 151 | 1969 | 48.6 | 60.5 | 37.0 | 68.1 | 93.4 | 47.9 |
| ICD-8 | 151 | 1970 | 47.3 | 58.6 | 36.5 | 65.3 | 88.9 | 46.5 |
| ICD-8 | 151 | 1971 | 47.4 | 58.3 | 36.8 | 64.2 | 87.4 | 45.8 |
| ICD-8 | 151 | 1972 | 47.2 | 58.6 | 36.3 | 63.2 | 87.1 | 44.5 |
| ICD-8 | 151 | 1973 | 46.9 | 57.7 | 36.4 | 61.9 | 85.0 | 44.0 |
| ICD-8 | 151 | 1974 | 46.1 | 57.0 | 35.5 | 59.9 | 83.0 | 42.2 |
| ICD-8 | 151 | 1975 | 44.8 | 55.6 | 34.4 | 56.9 | 79.4 | 39.8 |
| ICD-8 | 151 | 1976 | 44.6 | 55.2 | 34.3 | 55.6 | 77.6 | 38.9 |
| ICD-8 | 151 | 1977 | 44.2 | 54.3 | 34.4 | 53.9 | 74.9 | 38.0 |
| ICD-8 | 151 | 1978 | 43.3 | 53.5 | 33.4 | 51.6 | 72.3 | 36.1 |
| ICD-9 | 151 | 1979 | 43.8 | 54.1 | 33.8 | 51.1 | 71.8 | 35.7 |
| ICD-9 | 151 | 1980 | 43.4 | 53.9 | 33.2 | 49.4 | 69.9 | 34.1 |
| ICD-9 | 151 | 1981 | 42.8 | 53.6 | 32.3 | 47.6 | 68.0 | 32.4 |
| ICD-9 | 151 | 1982 | 41.5 | 51.7 | 31.7 | 45.0 | 64.1 | 30.9 |
| ICD-9 | 151 | 1983 | 41.6 | 52.4 | 31.1 | 43.9 | 63.3 | 29.5 |
| ICD-9 | 151 | 1984 | 41.7 | 52.5 | 31.1 | 42.9 | 62.2 | 28.8 |
| ICD-9 | 151 | 1985 | 40.7 | 51.1 | 30.6 | 40.8 | 59.1 | 27.5 |
| ICD-9 | 151 | 1986 | 39.9 | 50.7 | 29.5 | 38.8 | 56.9 | 25.7 |
| ICD-9 | 151 | 1987 | 39.8 | 50.6 | 29.3 | 37.5 | 55.2 | 24.7 |
| ICD-9 | 151 | 1988 | 39.3 | 50.0 | 29.0 | 36.0 | 53.1 | 23.7 |
| ICD-9 | 151 | 1989 | 39.4 | 50.3 | 28.8 | 35.1 | 51.9 | 22.9 |
| ICD-9 | 151 | 1990 | 38.7 | 49.6 | 28.1 | 33.4 | 49.5 | 21.6 |
| ICD-9 | 151 | 1991 | 38.9 | 50.1 | 28.1 | 32.6 | 48.7 | 20.9 |
| ICD-9 | 151 | 1992 | 38.9 | 50.3 | 27.9 | 31.6 | 47.4 | 20.1 |
| ICD-9 | 151 | 1993 | 38.2 | 49.4 | 27.5 | 30.1 | 45.2 | 19.2 |
| ICD-9 | 151 | 1994 | 38.5 | 50.2 | 27.2 | 29.5 | 44.7 | 18.4 |
| ICD-10 | C16 | 1995 | 40.3 | 52.6 | 28.5 | 29.8 | 45.4 | 18.5 |
| ICD-10 | C16 | 1996 | 40.2 | 53.0 | 28.0 | 28.9 | 44.3 | 17.6 |
| ICD-10 | C16 | 1997 | 39.8 | 52.6 | 27.5 | 27.7 | 42.6 | 16.8 |
| ICD-10 | C16 | 1998 | 40.5 | 53.6 | 27.9 | 27.3 | 42.1 | 16.4 |
| ICD-10 | C16 | 1999 | 40.4 | 53.4 | 27.9 | 26.4 | 40.8 | 15.9 |
| ICD-10 | C16 | 2000 | 40.3 | 53.3 | 27.8 | 25.5 | 39.1 | 15.3 |
| ICD-10 | C16 | 2001 | 39.7 | 52.4 | 27.5 | 24.2 | 37.1 | 14.6 |
| ICD-10 | C16 | 2002 | 39.1 | 51.6 | 27.1 | 23.0 | 35.3 | 13.8 |
| ICD-10 | C16 | 2003 | 39.3 | 52.2 | 27.0 | 22.4 | 34.5 | 13.2 |
| ICD-10 | C16 | 2004 | 40.1 | 53.3 | 27.4 | 22.2 | 34.2 | 13.2 |
| ICD-10 | C16 | 2005 | 39.9 | 53.0 | 27.4 | 21.2 | 32.7 | 12.5 |
| ICD-10 | C16 | 2006 | 40.0 | 53.2 | 27.4 | 20.7 | 31.9 | 12.0 |
| ICD-10 | C16 | 2007 | 40.1 | 53.9 | 27.0 | 20.0 | 31.1 | 11.5 |
| ICD-10 | C16 | 2008 | 39.8 | 53.7 | 26.6 | 19.3 | 30.0 | 11.0 |
| ICD-10 | C16 | 2009 | 39.8 | 53.4 | 26.7 | 18.7 | 29.0 | 10.7 |
| ICD-10 | C16 | 2010 | 39.7 | 53.5 | 26.5 | 18.1 | 28.2 | 10.2 |
| ICD-10 | C16 | 2011 | 39.5 | 53.3 | 26.3 | 17.6 | 27.4 | 9.9 |
| ICD-10 | C16 | 2012 | 39.0 | 52.5 | 26.2 | 16.9 | 26.1 | 9.6 |
| ICD-10 | C16 | 2013 | 38.7 | 52.3 | 25.8 | 16.3 | 25.2 | 9.2 |
| ICD-10 | C16 | 2014 | 38.2 | 51.6 | 25.5 | 15.6 | 24.1 | 9.0 |
| ICD-10 | C16 | 2015 | 37.2 | 50.5 | 24.7 | 14.8 | 22.9 | 8.3 |
| ICD-10 | C16 | 2016 | 36.4 | 49.0 | 24.4 | 14.0 | 21.6 | 8.0 |
| ICD-10 | C16 | 2017 | 36.3 | 49.0 | 24.2 | 13.5 | 20.9 | 7.6 |
| ICD-10 | C16 | 2018 | 35.6 | 47.7 | 24.1 | 12.9 | 19.7 | 7.4 |
| ICD-10 | C16 | 2019 | 34.7 | 46.6 | 23.4 | 12.2 | 18.7 | 7.0 |

**Abbreviation.** ICD, International Classification of Diseases.

**Supplementary Table 2. Expected mortality reduction of screening and relative risk of no screening for gastric cancer death according to the effectiveness and the proportion of endoscopy screening.**

|  |  | **Expected mortality reduction of gastric cancer screening** | | | **Relative risk for gastric cancer death in no screening** | | |
| --- | --- | --- | --- | --- | --- | --- | --- |
|  |  |  |  |  |  |  |  |
|  |  | **Mortality reduction of endoscopy screening** | | | | | |
|  |  | **0.20** | **0.30** | **0.60** | **0.20** | **0.30** | **0.60** |
| **Proportion of endoscopy screening** | **0.00** | 0.50 | 0.50 | 0.50 | 2.00 | 2.00 | 2.00 |
|  | **0.10** | 0.47 | 0.48 | 0.51 | 2.13 | 2.08 | 1.96 |
|  | **0.20** | 0.44 | 0.46 | 0.52 | 2.27 | 2.17 | 1.92 |
|  | **0.30** | 0.41 | 0.44 | 0.53 | 2.44 | 2.27 | 1.89 |
|  | **0.40** | 0.38 | 0.42 | 0.54 | 2.63 | 2.38 | 1.85 |
|  | **0.50** | 0.35 | 0.40 | 0.55 | 2.86 | 2.50 | 1.82 |
|  | **0.60** | 0.32 | 0.38 | 0.56 | 3.13 | 2.63 | 1.79 |
|  | **0.70** | 0.29 | 0.36 | 0.57 | 3.45 | 2.78 | 1.75 |
|  | **0.80** | 0.26 | 0.34 | 0.58 | 3.85 | 2.94 | 1.72 |
|  | **0.90** | 0.23 | 0.32 | 0.59 | 4.35 | 3.13 | 1.69 |
|  | **1.00** | 0.20 | 0.30 | 0.60 | 5.00 | 3.33 | 1.67 |

**Note.** Expected mortality reduction of gastric cancer screening changed according to the effectiveness and the proportion of endoscopy and fluoroscopy screenings and ranged from 0.20 (80% mortality reduction) to 0.60 (40% mortality reduction). The relative risk of no screening to gastric cancer screening for gastric cancer death was estimated from 1.67 to 5.00.
